# OTSun, a python package for the optical analysis of solar-thermal collectors and photovoltaic cells with arbitrary geometry

**DOI:** 10.1371/journal.pone.0240735

**Published:** 2020-10-14

**Authors:** Gabriel Cardona, Ramon Pujol-Nadal

**Affiliations:** 1 Departament de Ciències Matemàtiques i Informàtica, Universitat de les Illes Balears, Palma de Mallorca, Spain; 2 Institut des Sciences de l’Évolution, Université de Montpellier, Montpellier, France; 3 Departament d’Enginyeria Industrial i Construcció, Universitat de les Illes Balears, Palma de Mallorca, Spain; Oak Ridge National Laboratory, UNITED STATES

## Abstract

Ray tracing software systems are commonly used to analyze the optics of solar energy devices, since they allow to predict the energy gains of devices in real conditions, and also to compare them with other systems constantly emerging in the market. However, the available open-source packages apply excessive simplifications to the model of light-matter interaction, making that the optical behaviour of the systems can not be properly characterized, which in turn implies disagreements between physical experiments and computer simulations. We present here the open source python package OTSun, which applies the Fresnel equations in their most general form, without further simplifications, and is suitable for the simulation of both solar-thermal and photovoltaic systems. The geometrical objects used in this package are created using the parametric 3D modeler FreeCAD, which is also a free and open source program and allows for the construction of arbitrary geometries that can be analyzed with OTSun. These, and other software capabilities, make OTSun extremely flexible and accurate for the optical analysis of solar devices with arbitrary geometry. Additionally, OTSun has a companion webtool, OTSunWebApp, that allows for the usage of certain features of the package without the need to install anything locally. We also show here two numerical experiments that we performed in order to validate the model and implementation: The analysis of the optical efficiency of a Linear Fresnel Reflector (with moving objects), and of a second surface mirror (with variable wavelengths). In each case, the numerical computations had deviations of less than 0.25% from reference models (either computed with another program or with exact formulas).

## Introduction

There is a great variety of solar energy technologies [1], which, depending on the type of energy conversion, can be classified into two large groups: solar-thermal and photovoltaic technologies. The first one is intended to transfer heat, usually to a working fluid, that can be later converted into electricity. The second one is intended to directly generate electricity. Moreover, there are also mixed solar technologies (PVT) that perform both types of energy conversion. Furthermore, and regardless of the solar collector technology, the solar energy device can also be considered as an optical system, designed to maximize the absorption of solar radiation. Thus, the optical characterization of such devices is the key underpinning for improving solar energy conversion technologies [2].

In addition, with the increase of CPU and GPU capacities, simulations based on ray tracing of light are the most suitable techniques to obtain accurate results, since they allow for considering realistic configurations, with complex geometries and novel materials, and also enable to model different types of light sources [3]. In fact, the Monte Carlo Forward Ray Tracing (MCFRT) is the most used method for ray tracing simulations. It consists on applying the geometrical optics equations to the rays emitted from a light source to determine its interaction with an optical system (the solar energy device). At the end, the more realistic they are the optical models implemented in the MCFRT, the more diversity of solar energy materials can be considered for the optical system construction. This is the case of implementing the optical Fresnel equations to determine the path trajectory of the rays traveling through the system having into account: the ray wavelength, the light polarization, the complex refraction index and other physical properties of materials. With this method, several interesting parameters, such as the optical efficiency, flux irradiation and charge carrier generation, can be accurately determined. It is important to mention that rigorous optical models are needed to elucidate the optical behavior of a solar optical system.

Focusing on solar thermal applications, a review of existing optical simulation tools used for Solar Central Receiver Systems (SCRS) was recently presented in [3]. In this review, a total of 18 different software bundles have been analyzed. At present, only three of them, Tonatiuh [4], HOpS [5] and SolTrace [6], are licensed as open source. Another recent study focused in solar thermal applications was realized in the context of IEA/SHC Task 49 “Solar Heat Integration in Industrial Processes” [7], where a round-robin ray tracing software comparison for Linear Focusing Solar Collectors (LFSC) was done. In this study, the main differences between the software packages under study were found in how they model the angular dependency of the optical properties of the materials. As a consequence, different modeling options by different software tools produced different values of optical efficiency. The main reason is that they implement simplified models of optical Fresnel equations, instead of implementing them in its generic form.

On the photovoltaic applications side, optical simulation tools based on MCFRT for solar cells are also used [8]. While there are many proprietary software packages, for example, CROWM [9], actually, to the best knowledge of the authors, Scientrace [10] is the only open-source ray tracing software focused on photovoltaic cells [11].

It should be noted that some of the commercial software packages can be used for both solar-thermal and photovoltaic cells technologies, but no open source software has been found that applies to both technologies. Related to this, we mention here some proprietary packages that apply Fresnel equations: OptiCAD [12], ZEMAX [13], ASAP [14], TracePro [15], and COMSOL [16]. In all of them, the optical efficiency and radiance flux can be determined, among others parameters. However, we want to emphasize the importance of having open source software, since this allows for the inspection, modification, and implementation of new capabilities by the scientific community, which gives transparency and added value to the tool.

In this setting, our contribution with the development of OTSun is twofold. On the one hand, we cover the absence of open source software that is focused on both solar technologies (thermal and photovoltaic). On the second hand, we implement the Fresnel equations in their most general form, without further simplifications, avoiding the aforementioned problem reported in [7]. The OTSun package uses FreeCAD [17], a free 3D CAD system, in order to model the geometry of the optical systems to be analyzed, and also uses its API to compute the intersections of rays with the scene. FreeCAD is open sourced and permits to work with open file formats such as STEP, STL, DXF, and OBJ.

OTSun is a python package, open sourced under the MIT license. It can be obtained from the public repository https://github.com/bielcardona/OTSun, and can also be installed directly from the Python Package Index (PyPI) by calling pip install otsun.

We also remark the existence of the web tool OTSunWebApp, publicly available at http://otsun.uib.es, which has been developed to allow for making experiments without having to install anything. Some tutorials for this tool can be found in [18].

The aim of this paper is to give a full overview of the OTSun package. In Section Background, we give a brief account of the theory of geometrical optics and describe the algorithm we have used as a basis for our model. Section Implementation gives an overall view of the implementation of the different modules in the OTSun package. In Section The Model validation, we exhibit some experiments we have performed to validate our models and implementations. We conclude this manuscript with some final remarks in Section Conclusion.

## Background

The so-called ray tracing method is used to calculate the trajectory of light rays through systems composed by objects with different geometrical and optical properties. In the framework of solar energy, this implies that the optical efficiency of systems can be assessed by computer simulation. Two major elements are required for this purpose. First, a mathematical theory of optics. Second, an algorithm for the simulation of pathways of rays emitted in the system. These two main elements, which constitute the theoretical basis of OTSun, are described in the following subsections.

### Geometrical optics

Geometrical optics is a model that describes the propagation of light in terms of rays (see [19]). This model can be figured out from approximate solutions of Maxwell’s equations and is valid whenever the waves of light are propagated across and around objects with dimensions greater than the wavelength. Hence, ray theory does not cover phenomena such as interference and diffraction, but notice that OTSun is supplemented by the 1D transform matrix method (TMM) to consider interference in thin films (see item PolarizedThinFilm in Section The Material class).

The first main ingredient in this model is given by the Lambert-Beers law, which states that the initial and final energies (*E*_0_ and *E*, respectively) of a ray with wavelength λ traveling a distance ℓ inside an optical medium are related by the equation

$$\begin{array}{c} E=E_{0}e^{-\alpha (\lambda )\ell }, \end{array}$$ (1)

where *α*(λ) = 4*πk*(λ) is the absorption coefficient of the medium.

The second main ingredient establishes what happens when a ray hits a surface delimiting two different optical media. In this case, different phenomena can take place, since the ray may get absorbed, reflected or transmitted (or a combination of those). In either case, this models determines the ray direction in the new medium ${\vec{v}}_{2}$ in terms of the initial ray direction ${\vec{v}}_{1}$ and $\vec{n}$, the unit vector normal to the surface at the point of incidence, and pointing towards the first medium. The vector ${\vec{v}}_{1}$ also characterizes the incidence angle *θ*_1_ by means of the equation $\cos\theta_{1}=-{\vec{v}}_{1}\cdot \vec{n}$. Analogously, ${\vec{v}}_{2}$ characterizes the reflection/refraction angle *θ*_2_ via $\cos\theta_{2}=\pm {\vec{v}}_{2}\cdot \vec{n}$, where the sign is positive in case of reflection and negative in case of refraction.

In case of reflection, the new ray direction is given by the law of reflection:

$$\begin{array}{c} {\vec{v}}_{2}={\vec{v}}_{1}+2\cos\theta_{1}\vec{n}. \end{array}$$ (2)

If the ray is refracted (transmitted) the new ray direction ${\vec{v}}_{2}$ is then given by the Snell’s law:

$$\begin{array}{c} {\vec{v}}_{2}=r{\vec{v}}_{1}+(r\cos\theta_{1}-\sqrt{1-r^{2}\sin^{2}\theta_{1}})\vec{n}. \end{array}$$ (3)

In this equation, r=Re(n˜1/n˜2), where ${\tilde{n}}_{i}\left(\lambda \right)=n_{i}\left(\lambda \right)-ik_{i}\left(\lambda \right)$ (for *i* = 1, 2) is the complex refractive index in each of the two media. Finally, Fresnel’s equations give the reflectance *R* and transmittance *T* rates for the energy of incident rays as follows (we have used the formalism exposed in [20, Ch. 2]):

$$\begin{array}{c} R=(\frac{\eta_{1}-\eta_{2}}{\eta_{1}+\eta_{2}}){\left(\frac{\eta_{1}-\eta_{2}}{\eta_{1}+\eta_{2}}\right)}^{*}, \end{array}$$ (4)

T=4η1Re(η2)(η1+η2)(η1+η2)*, (5)

where *η*_1_ and *η*_2_ are the tilted optical admittances of the two optical media, and they depend on the polarization of the incident light:

$$\begin{array}{c} \eta_{i}=\{\begin{array}{cc} \frac{{\tilde{n}}_{i}\left(\lambda \right)\cos\theta_{i}}{Z_{0}}, & \text{for}\;s-\text{polarization},\\ \frac{{\tilde{n}}_{i}\left(\lambda \right)}{Z_{0}\cos\theta_{i}}, & \text{for}\;p-\text{polarization}, \end{array} \end{array}$$ (6)

being $Z_{0}=\sqrt{\mu_{0}/\in_{0}}$ the optical impedance of free space.

### The ray tracing algorithm

**Fig 1:**
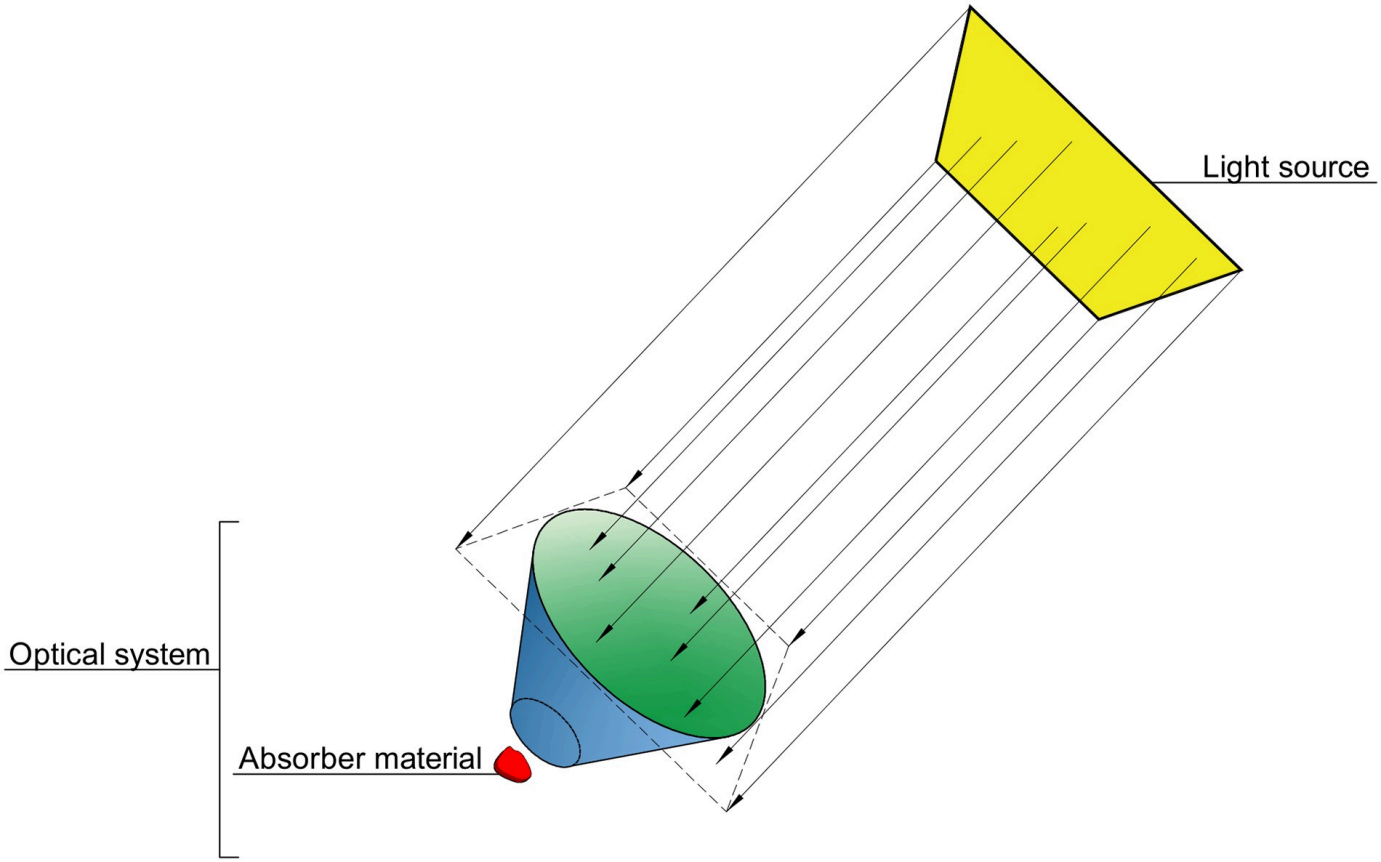
Overview of the key elements of the ray tracing procedure. The scene is composed by the optical system, formed by FreeCAD volume and surface elements. On the scene, at least one material has the functionality of light absorption. The light source emits rays to the scene.

The key ingredients for any ray tracing simulation are shown in Fig 1. The light source is the geometric region from where rays are emitted to the scene. The optical system is formed by different elements that interact with the rays, some of which are made of absorber materials that capture energy. In our implementation, the optical system is composed by surface and volume elements generated by FreeCAD, and each of them must be associated to an optical material (otherwise it will not interact with light rays). Two kinds of special materials are implemented for defining the absorber material: thermal materials (for solar-thermal collectors) and the PV materials (the active materials of photovoltaic cells). See Section Implementation for details on how we implement all these ingredients.

**Fig 2:**
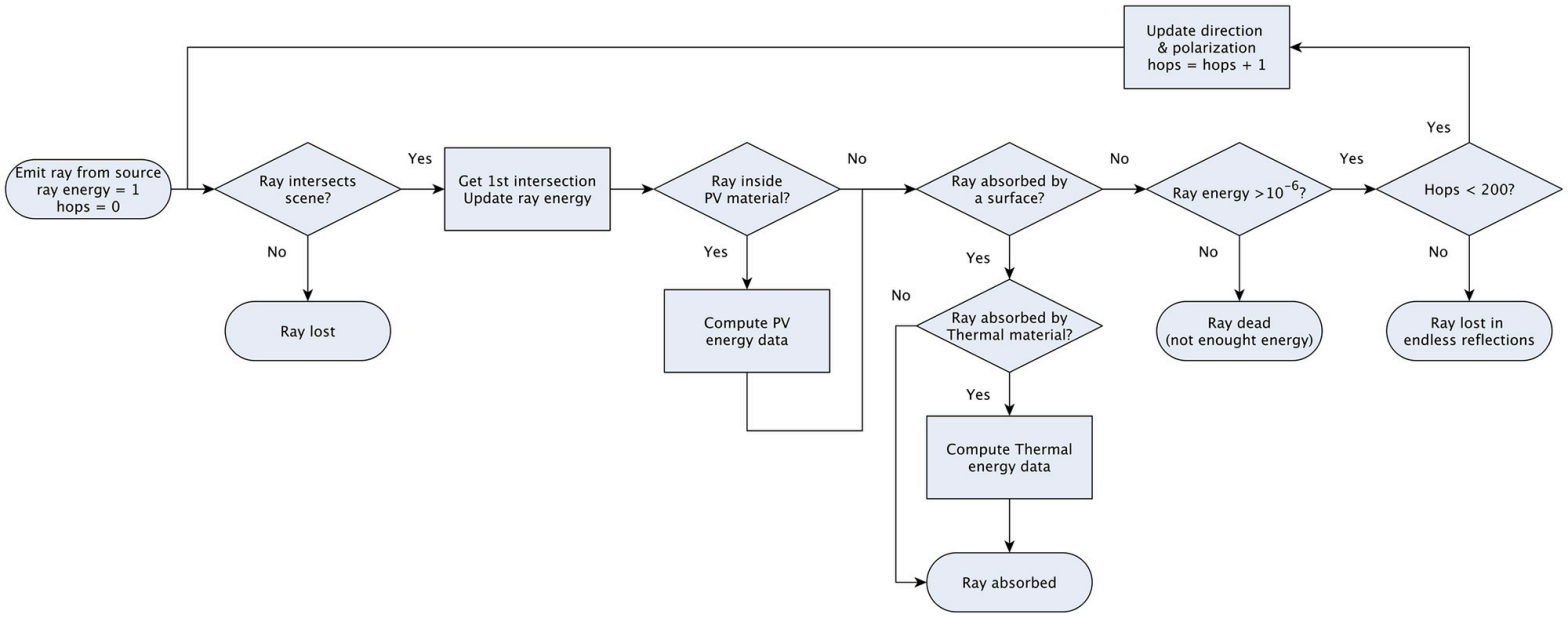
Overview of the sequential ray tracing algorithm.

An overview of the algorithm for the tracing of each single ray is shown in Fig 2. The path followed by a ray is composed of several “hops”, corresponding to different rectilinear segments where the ray moves inside a fixed optical medium. The algorithm starts with the simulation of the emission of a ray from the light source. The point of emission, direction, polarization and wavelength are determined by the light source, while the energy is normalized to be unitary. Then, we sequentially simulate hops, determined by the interaction of the ray with the scene as follows. First, we find the closest intersection of the simulated ray with the scene; in case no intersection is found, then the ray has exited the scene and the tracing is finished. In case we find such an intersection, we update the energy level of the ray at this point, since it could have been traveling inside a dissipative medium during the current hop; also, and in the case that this medium is a “PV material”, we compute the collected energy for future computations. Next, we determine if the ray has been absorbed at the point of intersection, either by having found a “Thermal material” (and in this case we compute the absorbed energy for future computations) or some other absorbent material; in any case, the ray tracing is finished. Likewise, if the energy level of the ray is below a threshold (by default 10^−6^), the ray is assumed to have disappeared and the tracing is finished too. Furthermore, we check if the number of hops has reached its maximum (200 hops by default), in which case we determine that the ray has been caught in a loop and stop the simulation. Otherwise, and since at this point the tracing of the ray has to continue, we simulate the optical effect (either reflection or refraction) of the materials on the ray to compute its new direction and polarization, and proceed to the simulation of the next hop.

## Implementation

The python package OTSun (imported as otsun) is composed by different modules, some of them collecting helper functions that implement both mathematical and optical methods, and other ones built around each of the main classes that define the functionality of the package. To ease the reading of this section, where we shall be dealing with classes and instances of those classes, whenever a class (say Scene or LightSource) is considered, the downcase version of their identifiers (scene and light_source in our example) will indicate instances of those classes. Except for those classes defined by FreeCAD (like Part.Face or Base.Vector), we refer to the corresponding section in this manuscript for the definition and initialization options for each of these classes. For instance, in Section The Experiment class we explain how to create an experiment (an instance of the Experiment class), giving an scene and a light_source, which are instances of Scene and LightSource, described in Sections The Scene class and The LightSource class, respectively.

A typical use of the package involves the creation of an experiment, which specifies the solar optics experiment to be run, defined by certain data that includes a scene and a light_source. The scene holds the data of all the different objects that interact with light rays, included in a FreeCAD document [17], and where each of them has a material associated describing its optical behaviour. Eventually, some objects may have movements, implemented by a multi_tracking, and in such a case these elements will be moved to maximize the absorbed energy. When the experiment is run, the light_source creates rays, which interact with the scene until they either leave the scene or are absorbed, and in this last case the collected energy (among other data) is stored for future analysis.

We comment now the main classes that have been implemented, together with its basic functionality. The complete documentation of the API can be found at https://otsun.readthedocs.io.

### The Experiment class

An experiment is initialized giving the parameters that define it: An scene and a light_source that describe the physical environment where the experiment takes place, and the number of rays that have to be simulated. The execution of the experiment is launched with the experiment.run() method, and once it is finished, the information that has been recollected is found in instance variables like experiment.captured_energy_Th and experiment.captured_energy_PV, that give the overall thermal and photovoltaic (respectively) energy that has been collected by the active materials found in the scene.

### The Scene class

Instances of the class Scene hold the data used to describe the physical objects present in an experiment, stored in three main variables, faces, solids and materials. Each object in the array faces (resp. solids) is a Part.Face (resp. Part.Solid) object of FreeCAD that represents a surface (resp. volume) that can affect the propagation of a ray incident with it. The dictionary materials assigns to each face or solid a material that describes its optical properties.

Such a scene is initialized with an array objects, all whose elements are instances of Part.Feature, and typically they are all the objects included in a FreeCAD document. The assignation of materials to each object is done by looking at its label. Namely, if an object obj in a FreeCAD document has a label of the form “Label(mat_name)”, then the assigned material scene.materials[obj] will be the Material instance mat such that mat.name is mat_name.

For instance, the file ParabolicTrough.FCStd in the public repository https://github.com/bielcardona/OTSunSuppMat (see also Section The MultiTracking class) contains a model prepared to analyze a parabolic trough collector. The parabolic mirror (in blue) is made by extruding a parabolic segment, and its label is Parabolic_reflector(Mir1). It means that when imported with OTSun, it will have an associated material whose parameter name is Mir1. In turn, this material has to be properly defined (see Section The Material class) so that it behaves as a mirror. Other elements in this model are the central cylindrical surface (in red), labeled Cylindrical_absorber(Abs1), and its covering (in green), labeled Tube_glass(Glass1); hence, materials named Abs1 and Glass1 have to be defined as an absorber surface and as a transparent volume material, respectively (see Section The Material class).

### The LightSource class

Instances of the class LightSource are used to model the source of rays in an experiment. There are many parameters that define its behaviour, like its emitting_region, describing the physical location of the source of the rays to be emitted, and its light_spectrum and direction_distribution, describing respectively the distribution of wavelengths and directions of the rays to be emitted.

The parameter light_spectrum can either be a constant, meaning that all rays will be emitted with the same specified wavelength (given in nanometers), or a cumulative distribution function (CDF) *F*(λ) which is defined by interpolation on the discrete values (λ_*i*_, *F*(λ_*i*_)) stored in an array ((λ_1_, λ_2_, …), (*F*(λ_1_), *F*(λ_2_) …).

The emitting_region has to be an instance of any class that implements the method random_point(), which returns a random point from where a ray will be emitted, and has an attribute main_direction, giving the direction of the emitted ray. For convenience, the class SunWindow implements such an emitting region as a plane rectangle *Π* in the space, orthogonal to a fixed direction $\vec{u}$, and such that all the objects in the scene are contained in the rectangular semi-prism $\left\{\Pi +\xi \vec{u}|\xi \geq 0\right\}$.

The parameter direction_distribution can either be None (meaning that the emitted rays are emitted in the main direction) or an instance of a class that implements the method angle_distribution(), giving a random angle (in degrees) of deviation for the emitted ray with respect to the main direction $\vec{u}$. For convenience, the class BuieDistribution implements such deviation according to the Buie distribution [21], determined by its circumsolar ratio (CSR), which is a parameter of the class.

### The Ray class

Instances of the class Ray model light rays, which are emitted by a light_source. A ray is initialized giving its initial optical_state, as well as the scene where it will travel. Instances of OpticalState gather some relevant information of a light ray at a given moment, like its direction, polarization and material, giving, respectively, the direction and polarization vector of the ray, and the material (optical medium) where it is traveling. When the method ray.run() is called, the propagation of the ray inside the scene starts to be simulated. A simplified version of the iteration process is (see also Section The ray tracing algorithm):

1. Find the closest intersection of ray with objects in scene.
2. If no intersection is found, the ray is lost and the simulation is finished.
3. If the first intersection is with an object having a determined material, then the method material.change_of_optical_state() is called (with different parameters that determine how the ray hits the material), which decides if the ray is reflected or refracted (and gives the next optical state) or that the ray has been absorbed by some active optical element.
4. If the ray has been reflected or refracted, go to step 1. Otherwise, the simulation is finished.

### The Material class

The Material class is the most complex of all the classes implemented in OTSun, since there are many kinds of materials, and their optical properties need to be explicitly defined. There are two main subclasses, SurfaceMaterial and VolumeMaterial, corresponding, respectively, to materials that can be assumed to be two-dimensional (like first surface mirrors and selective absorbers) or not (like glasses, second surface mirrors, PV active materials, thin films,…). Any material has an important property, material.name, indicating how it will be called when identifying objects in a scene as explained in Section The Scene class. The physical properties of a material are encoded in material.properties, a dictionary whose contents depend on the kind of material.

Any user willing to use his own materials in his experiments can subclass SurfaceMaterial or VolumeMaterial to adapt the contents of material.properties, which implement the specific properties of the materials. The user must override the method material.change_of_optical_state() to implement the computation of how the interaction with the material changes the optical state (direction, polarization, etc.) of a ray.

Additionally, since it is interesting to store externally the properties of materials, the method material.to_json() and the class method SubclassedMaterial.load_from_json(info) should be implemented. The first one must convert any information stored in material.properties into a serializable dictionary, and the second one must use this dictionary to reconstruct the material.properties dictionary.

#### The VolumeMaterial class

Instances of VolumeMaterial represent the optical properties of physical objects whose depth is not negligible, like glasses or PV active materials, where the ray energy attenuation is determined by the Beer–Lambert law (1). In this case, the method material.change_of_optical_state() is generically implemented using the law of reflection (2), Snell’s law (3), and Fresnel’s Eqs (4)–(6), but any user could subclass it and implement some other optical behaviour of the material.

Some subclasses of this class are provided, so that materials appearing usually in the field of solar collectors can be used without further implementation. For example:

- SimpleVolumeMaterial, representing a material with constant optical parameters (refraction index and absorption coefficient, given in mm^−1^).
- WavelengthVolumeMaterial, where the index of refraction is complex ($\tilde{n}=n-i\kappa$) and depends on the wavelength of the ray. These values are computed by interpolation from data given in tabulated form with rows (λ, *n*(λ), *κ*(λ)). Note that the imaginary part of the refractive index is the so called the extinction coefficient, and the absorption coefficient is calculated as *α* = 4*πκ*/λ. The wavelengths are given in nanometers.
- PolarizedThinFilm, which represents a thin layer, such as an optical coating, where the thickness and light coherence (that enables interference) can not be considered as negligible in the simulation. The data values are given in tabulated form with rows (λ, *θ*, *R*_*s*_(λ, *θ*), *R*_*p*_(λ, *θ*), *T*_*s*_(λ, *θ*), *T*_*p*_(λ, *θ*)), where *θ* is the incidence angle, *R* and *T* denote the power reflection and transmission coefficients respectively, and sub-indexes *s* and *p* denote respectively the perpendicular and parallel ray polarization. Wavelengths are given in nanometers and incidence angles in degrees. We remark that it is precisely in this case where the ray equations are complemented by the so-called fully-coherent medium transfer matrix formalism (TMM).
- PVMaterial, which represents the active material in photovoltaic cells such as semiconductors or any other material with that functionality. This is the case of the “PV material” exposed in Section The ray tracing algorithm. The photo-absorption in such materials is characterized by their extinction coefficient. The values of the index of refraction $\left(\tilde{n}=n-i\kappa \right)$, which depends on the light wavelength, are given in tabulated form as in the WavelengthVolumeMaterial case.

#### The SurfaceMaterial class

Any surface_material represents a two-dimensional physical object, in the sense that its third dimension is negligible, or simply that its behaviour does not depend on it. Examples of these objects are front surface mirrors, selective absorbers, metallic coatings,…. In a first approximation, the interaction of a ray with such a material can result in a reflection, an absorption or a transmittance, each with a given probability that may depend on the wavelength of the ray and are stored in the dictionary p = material.properties. Hence, material.change_of_optical_state() generically implements these different phenomena. This behaviour is also affected by other properties of the material, like the booleans:

- p['lambertian_material'], indicating that, in the case of reflection, the direction of the reflected ray should be a random vector, instead of that computed using the law of reflection.
- p['thermal_material'], indicating that, in case of absorption, the energy is absorbed and processed, instead of lost in the material. This is the case of the “Thermal material” exposed in Section The ray tracing algorithm.

Some more specific materials are provided by subclassing SurfaceMaterial and overriding the change_of_optical_state() method. Some examples of these specific materials are:

- AbsorberTWModelLayer, represents a thermal absorber where its absorption depends on the incidence angle, *θ*, according to $\alpha =\alpha_{0}\{1-b{\left(\frac{1}{\cos\theta }-1\right)}^{c}\}$, see [22] for more details. The following data values are given: *α*_0_, *b*, *c*. In this case, the boolean property p['thermal_material'] is True.
- MetallicSpecularLayer, represents a metal surface, such as the silver coating in second surface mirrors. Fresnel equations are considered and its characterization is defined by the complex index of refraction $\left(\tilde{n}=n-i\kappa \right)$ depending on the light wavelength. The data values are given in tabulated form like in the WavelengthVolumeMaterial case.
- MetallicLambertianLayer, represents a metal surface where Fresnel equations are considered, but if the ray is reflected, a total diffuse reflection model with Lambertian scattering is used. In this material, the boolean property p['lambertian_material'] is True. Also, its characterization is defined by the complex index of refraction $\left(\tilde{n}=n-i\kappa \right)$ depending on the light wavelength. The data values are given in tabulated form like in the WavelengthVolumeMaterial case.
- PolarizedCoatingLayer, and its subclasses PolarizedCoatingReflectorLayer, PolarizedCoatingTransparentLayer, PolarizedCoatingAbsorberLayer, that represent thin layers such as optical coatings. The difference with the PolarizedThinFilm is that the thickness of such material is negligible. The data values are given as in the PolarizedThinFilm case. Depending on the role of the material, three cases are defined: reflector (no light transmission is possible), transparent (reflection, absorption and transmission are possible), and thermal absorber material (the boolean property p['thermal_material'] is True and no light transmission is possible). In each case, the parameters are given analogously to the case of PolarizedThinFilm.

### The MultiTracking class

The class MultiTracking is designed to implement movements of the active elements in a scene so that the rays emitted by a given light_source tend to be focused on a target (in case that the attribute target is set to a point) or tend to return it to the source (in case that the attribute is not set). That is, MultiTracking can be used either to orient the solar collector to the sun or to direct rays to a target, as happens with the segment mirrors of a Linear Fresnel Collector (LFR) or the heliostats in solar power tower plants.

Movements of elements are implemented by the helper class Joint, and its subclasses CentralJoint and AxialJoint. The former implements rotations around a given point in space (that is, with two degrees of freedom), while in the latter the rotations are around an axis (and hence with a single degree of freedom). Each kind of joint can be easily represented by a geometrical object in FreeCAD, either by a Vertex or an Edge with two points.

To describe the movement of a concrete element in the scene, one needs to associate to this object a joint, but since the goal is to direct the rays to a specified region, one also needs to specify the corresponding *principal vector*. Here, by the *principal vector*, we mean the direction that best approaches the normal of the mobile element. When multi_tracking.target is not set, the element will be moved so that this vector points to the source; otherwise, the movement will be computed so that a solar ray reflected on the plane normal to the *principal vector* and passing through the joint hits the point stored in multi_tracking.target.

We associate objects in the scene to joints using the following convention (see also Section The Scene class): Instead of giving to the object under consideration a label of the form “Label(mat_name)”, where mat_name is the identifier of the material of the object, we use a label of the form “Label(mat_name,joint_name,normal_name)” or “Label(mat_name,joint_name,normal_name,target_name)”, where joint_name is the label of the FreeCAD object that describes the joint (i.e. either a Vertex or a Edge), normal_name is the label of the FreeCAD Edge whose direction is the *principal vector* of the optical element, and target_name (if present) is the label of the FreeCAD object acting as target.

A multi_tracking is created by giving the scene (which includes the elements that describe the joints, together with their principal vectors and targets, if needed) and the light_source, a Base.Vector giving the main direction of the sun rays. Once it is created, the method target_tracking.make_movements() transforms the scene, rotating conveniently the elements, so that the scene behaves as explained above.

## Model validation

We discuss here two experiments showing how the results obtained with OTSun are compared with those obtained using other software tools, hence providing a validation for both our model and our implementation. A third validation example, related to the optical behaviour of perovskite solar cells, can be found in [2]. In each case, we have compared the results obtained by OTSun with a reference model using both the mean error (ME), so that we can determine if OTSun has an overall tendency to overestimate or underestimate the results, and the root mean square error (RMSE), to evaluate the global accuracy. The scripts and auxiliary files cited in this section can be found at https://github.com/bielcardona/OTSunSuppMat.

### First experiment

We have compared the computations of the optical efficiency of a Linear Fresnel Reflector (LFR) obtained using OTSun and Tonatiuh [4]. We have chosen this geometry due to its complexity, having mobile objects and using four different types of optical materials. The geometry of the LFR is composed by 11 parabolic mirrors of 500 mm width, a secondary CPC reflector with a maximum concentration of 1.66 truncated at 61.81 mm, and a flat receiver of 70 mm width placed 6 meters above the mirrors field. The parabolic mirrors track the sun with the purpose of reflecting the sunlight to a target located at 46.45 mm under the flat receiver. Each parabolic mirror has a focal length equal to the distance between its center position (which includes the rotation axis) and the mentioned target. The files LFR.FCStd and LFR.tnh contain the implementation of this geometry in FreeCAD and Tonatiuh, respectively. Additionally, the file LFR_output.FCStd shows the output given by OTSun with the simulation of some rays with a transversal incidence angle equal to 45 degrees. Since the optics implemented in Tonatiuh is not based on the Fresnel equations in all its generality, we took the decision to use materials with constant optical properties, so that we can use materials with the same properties in both programs. The file validation1.py contains the specification of the optical materials used in this example, as well as the code used to compute the optical efficiency.

**Fig 3:**
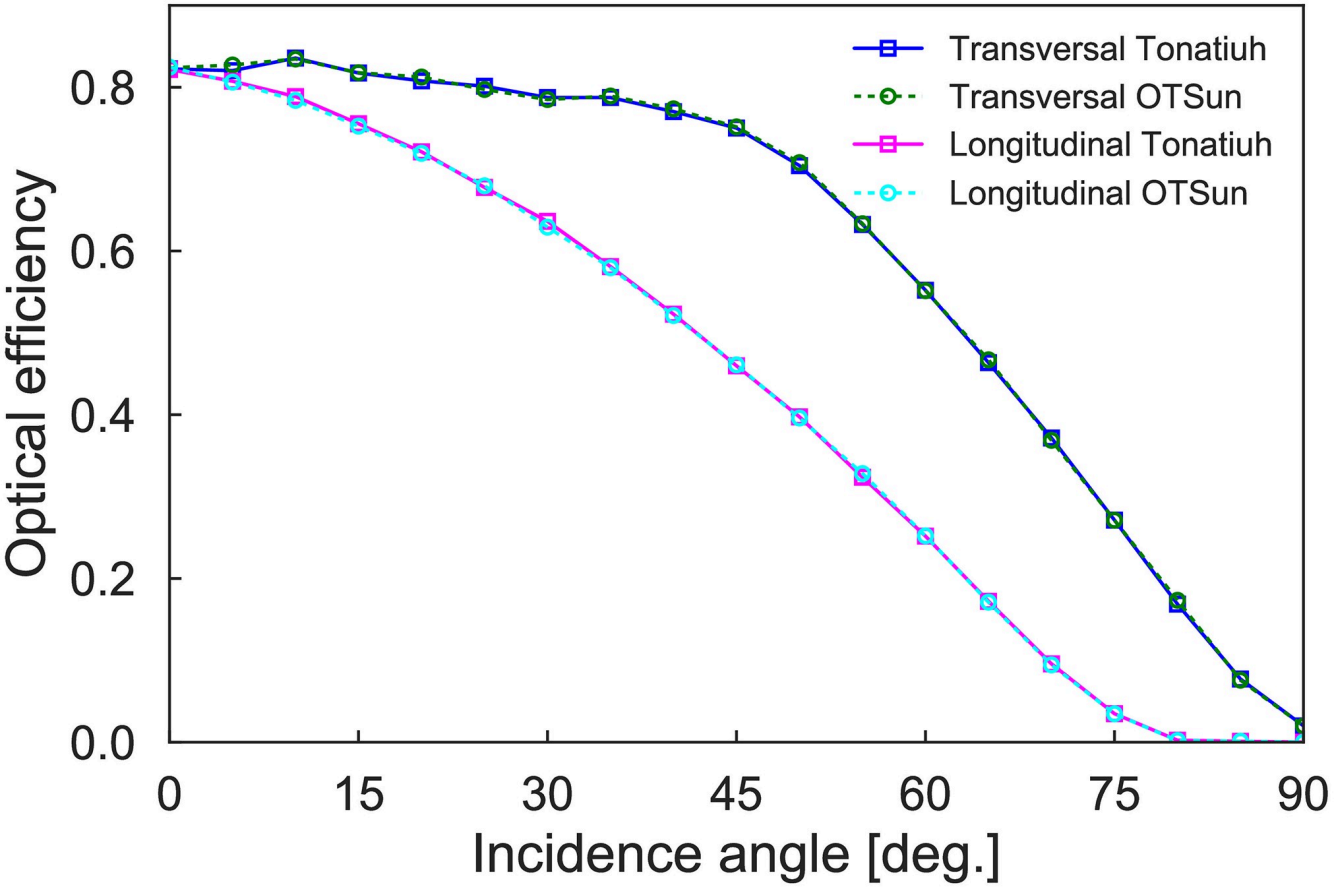
Optical efficiency at transversal and longitudinal planes of the analyzed LFR, as computed by OTSun and Tonatiuh.

In our computations (both with OTSun and Tonatiuh), we have simulated the emission of 100,000 sun rays per each sun position, using the Buie model approach [21] with a value of 0.05 for the circumsolar ratio. Fig 3 shows the optical efficiencies obtained with the two software packages using different angles for the sun position in both the transversal and longitudinal planes.

### Second experiment

We have determined the reflectance of a second surface mirror composed by a layer of 4 mm of borosilicate glass with a silver coating. The model can be found in the file mirror.FCStd, where it can be seen that we created a surface material formed by two layers to be put over the mirror. The outside layer is a transparent material, while the inner one is an ideal thermal material absorber. With this setting, the rays that reach the outer layer pass through it, and those that are later reflected by the mirror are collected by the absorber material, hence providing a measure of the optical efficiency of the mirror. This efficiency, divided by the cosine of the incidence angle of the light rays with respect to the normal vector of the mirror surface, gives the reflectance of the mirror. See the files mirror.FCStd and validation2.py for more details on this geometry and the script we have used to make the simulation with OTSun. Also, file mirror_output.FCStd shows the output produced by OTSun with some simulated rays drawn.

**Fig 4:**
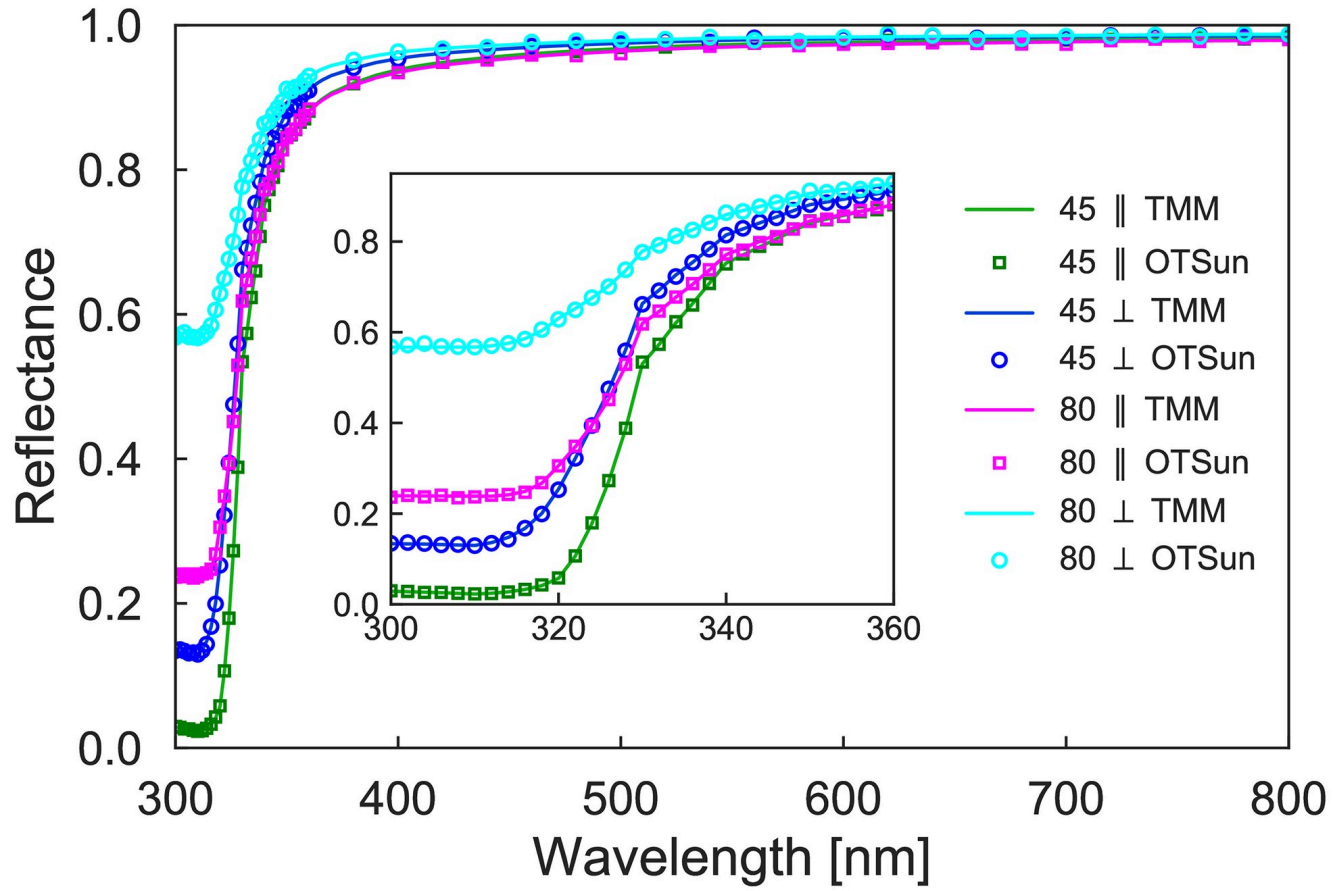
Reflectance of a second surface mirror at an incidence angle of 45 and 80 degrees, with parallel and perpendicular polarization, computed using the TMM and OTSun.

The optical properties of this configuration can also be determined by the analytic transform matrix method (TMM), as exposed in [23] and implemented in [24]. A comparison of the results obtained using these two methods is shown in Fig 4, where the reflectances are plot as a function of the wavelength using two different incidence angles (*θ* = 45° and *θ* = 80° degrees) and with parallel and perpendicular polarizations.

### Robustness of validations

To prove the robustness of the validations, we have computed the mean error (ME) and the root mean square error (RMSE) between the results obtained by OTSun and those obtained either by Tonatiuh or TMM. Table 1 summarizes these computations.

**Table 1:** Mean Error (ME) and Root Mean Square Error (RMSE) between OTSun simulations and the reference models.

Experiment	Case	ME	RMSE
First	Transversal	0.00130	0.0020
First	Longitudinal	−0.00983	0.0013
Second	*θ* = 45°, ∥ pol.	0.00035	0.0015
Second	*θ* = 45°, ⊥ pol.	−0.00019	0.0016
Second	*θ* = 80°, ∥ pol.	−0.00193	0.0024
Second	*θ* = 80°, ⊥ pol.	−0.00019	0.0025

One remark is due for these results. In the case of the second experiment, plotted in Fig 3, the optical efficiency in the longitudinal plane for angles greater than or equal to 80 degrees is nearly zero, since the sun is nearly in the direction of the axis of the mirrors and hence nearly no reflections are captured by the receiver. To avoid the spurious behaviour of the indicators due to values close to zero, we have opted for omitting the values corresponding to these angles in the computation of ME and RMSE.

From Table 1, we see that the ME takes both positive and negative values, which implies that OTSun does not present any tendency to overestimate or underestimate the results with respect to the reference models. From the result obtained by OTSun respect to the reference cases, we can see that OTSun presents RMSE values between 0.0013 and 0.0025 for the cases analyzed, which represents an error lower than 0.25%.

## Conclusion

Ray tracing software systems are commonly used to make optical analysis of solar energy devices. However, the available open source systems make excessive simplifications in the implementation of optics, causing limitations on their features. In this paper, we have presented the open source package OTSun. It is a Monte Carlo ray tracing python library, where the optics is implemented using the Fresnel optics equations in their most general form. The optical phenomena implemented in OTSun allow for dependence on wavelength, incidence angle, light polarization, interference, ray attenuation, complex refractive index, source spectrum and direction of emission, among others. OTSun also implements tracking movements, so that objects move to track the sun or to reflect the rays onto a specific target. The geometry of the optical systems used by OTSun is built with FreeCAD, which is also open source, and hence allows for the simulation of solar energy devices with arbitrary geometry. In addition, OTSun is accompanied by the webtool OTSunWebApp, that allows to make some simulations without the need to install anything locally.

We presented numerical simulations to validate the OTSun models and implementations. For this purpose, we made two different experiments. The first one is mainly devoted to testing the reflections and movements of optical elements, while the second one focuses on the full implementation of the Fresnel equations. The robustness of such validations is demonstrated, achieving deviations lower than 0.25% from the reference models, Tonatiuh and TMM, respectively.

We think that OTSun will become a valuable resource for the community of designers and researchers on solar optics. But not only for them: It has been demonstrated that OTSun is a software tool suitable to analyze opto-mechanic problems, due to its flexibility and that the optics equations are implemented in its most general form. To this end, and as our future work, we have in mind to develop modules for FreeCAD to simplify the construction of optical elements such as lenses, collimators, filters, etc., and solar energy devices such as LFRs, PTCs, solar power towers, PV cells, etc.

In its present form, OTSun is exclusively an optics simulator. Even with this limitation, and for the case of solar cells, we have implement functions to obtain the photocurrent if the internal quantum efficiency is given as input. A future work consists in integrating a solver for the carrier transport equations, to evaluate the capabilities of power electricity conversion of solar cells. This would provide in a single tool, the capability of considering both optical and electronic aspects of solar cells.
